# Herbicidal Activity of *Thymbra capitata* (L.) Cav. Essential Oil

**DOI:** 10.3390/molecules25122832

**Published:** 2020-06-19

**Authors:** Mercedes Verdeguer, Natalia Torres-Pagan, Marta Muñoz, Amira Jouini, Susana García-Plasencia, Pablo Chinchilla, Mónica Berbegal, Adele Salamone, Santo Agnello, Alessandra Carrubba, Luz Cabeiras-Freijanes, Lois Regueira-Marcos, Adela M. Sánchez-Moreiras, María Amparo Blázquez

**Affiliations:** 1 Instituto Agroforestal Mediterráneo (IAM), Universitat Politècnica de València, Camino de Vera s/n, 46022 Valencia, Spain; natorpa@upv.es (N.T.-P.); mmunoz@seipasa.com (M.M.); amjo@doctor.upv.es (A.J.); sgplasencia@hotmail.com (S.G.-P.); pabchial@gmail.com (P.C.); mobermar@etsia.upv.es (M.B.); 2 SEIPASA S.A. C/Ciudad Darío, Polígono Industrial La Creu naves 1-3-5, 46250 L’Alcudia, Valencia, Spain; 3 Dipartimento di Scienze Agrarie, Alimentari e Forestali, Università degli Studi di Palermo, Viale delle Scienze, Ed. 4, 90128 Palermo, Italy; alessandra.carrubba@unipa.it; 4 Consiglio per la Ricerca in Agricoltura e L’analisi dell’economia Agraria, Centro di Ricerca Difesa e Certificazione, Sede di Palermo, Viale Regione Siciliana Sud Est, 8669 Palermo, Italy; adele.salamone@crea.gov.it; 5 Consiglio per la Ricerca in Agricoltura e l’analisi dell’economia Agraria, Centro di Ricerca Difesa e Certificazione, Sede di Bagheria, S.S. 113-km 245.500, 90011 Bagheria (Palermo), Italy; santo.agnello@crea.gov.it; 6 Department of Plant Biology and Soil Science, Faculty of Biology, University of Vigo, Campus Lagoas-Marcosende s/n, 36310 Vigo, Spain; luzcabeirasfreijanes@gmail.com (L.C.-F.); lewis_coya@hotmail.com (L.R.-M.); adela@uvigo.es (A.M.S.-M.); 7 CITACA, Agri-Food Research and Transfer Cluster, Campus da Auga, University of Vigo, 32004 Ourense, Spain; 8 Departament de Farmacologia, Facultat de Farmàcia, Universitat de València, Avda. Vicent Andrés Estellés s/n, 46100 Burjassot, València, Spain

**Keywords:** weed control, natural herbicides, essential oils, *Thymbra capitata*, carvacrol, integrated weed management, bioherbicides

## Abstract

The bioherbicidal potential of *Thymbra capitata* (L.) Cav. essential oil (EO) and its main compound carvacrol was investigated. In in vitro assays, the EO blocked the germination and seedling growth of *Erigeron canadensis* L., *Sonchus oleraceus* (L.) L., and *Chenopodium album* L. at 0.125 µL/mL, of *Setaria verticillata* (L.) P.Beauv., *Avena fatua* L., and *Solanum nigrum* L. at 0.5 µL/mL, of *Amaranthus retroflexus* L. at 1 µL/mL and of *Portulaca oleracea* L., and *Echinochloa crus-galli* (L.) P.Beauv. at 2 µL/mL. Under greenhouse conditions, *T. capitata* EO was tested towards the emergent weeds from a soil seedbank in pre and post emergence, showing strong herbicidal potential in both assays at 4 µL/mL. In addition, *T. capitata* EO, applied by spraying, was tested against *P. oleracea*, *A. fatua* and *E. crus-galli*. The species showed different sensibility to the EO, being *E. crus-galli* the most resistant. Experiments were performed against *A. fatua* testing *T. capitata* EO and carvacrol applied by spraying or by irrigation. It was verified that the EO was more active at the same doses in monocotyledons applied by irrigation and in dicotyledons applied by spraying. Carvacrol effects on *Arabidopsis* root morphology were also studied.

## 1. Introduction

The family Lamiaceae is one of the most widely used source of spices, and includes medicinal plants endowed with strong antimicrobial and antioxidant properties [1,2,3,4]. Extracts from Lamiaceae have been reported to possess a wide range of biological activities, as well as phytochemical diversity [5]. There is a long history of controversies surrounding this family [6], resulting in a considerable number of recent efforts to re-evaluate existing classification by means of molecular phylogenetic analyses [7,8,9,10,11]. Among the most challenging taxa there is the subtribe Menthinae (Lamiaceae, Nepetoideae, Mentheae), which includes well known aromatic plants as peppermint, oregano, savory and thyme (see [12] for a comprehensive review). This led to an enormous number of names [13] with many synonyms under different generic names and, as a consequence, to a considerable taxonomic confusion. A genus that was previously considered rather isolated in this group is *Thymbra* Linnaeus, comprising four species of Mediterranean subshrubs [14,15]. The recent molecular phylogenetic analysis of both nuclear ribosomal and plastid markers [12] revealed that this genus had a distinct lineage, dissociated from both *Thymus* Linnaeus and *Satureja* Linnaeus [6].

*Thymbra capitata* (L.) Cav. is a Mediterranean species typically found in garrigues, dry slopes and Mediterranean pine forests, which grows between 0 and 600 m above sea level, and is considered a good ecological indicator of the dry Mediterranean area [16,17]. In Spain, *T. capitata* and, in general, *Thymus* species are commonly known as thyme, and are currently used as culinary herbs, as well as for ornamental, flavouring and medicinal purposes [18]. The essential oil (EO) from *T. capitata* is greatly appreciated and deals with a deep economic importance because of its biological properties. In the last ten years much research has been carried out on this species, enlightening the EO composition and its biological activities, such as antimicrobial [19,20,21,22,23], antifungal [24,25,26], and antioxidant [21,26,27]. Since EO components are responsible for the different EO biological activities, the knowledge of the EO’s composition, as well as of the factors related to its variability, is of outstanding relevance [28].

The composition of *T. capitata* EO from different origins has been thoroughly studied. *T. capitata* EOs from populations growing in Sicily [29,30], Sardinia [31,32], and Albania [33,34], always contained carvacrol (65.1–86.3%), and small amounts of thymol, usually below 1% [17]. EOs from different *T. capitata* populations from southern Puglia (Italy) were also analysed, detecting three chemotypes: thymol, carvacrol, and thymol/carvacrol [17]. Environmental factors influencing *T. capitata* EO composition were studied, allowing to assess that the carvacrol chemotype was only present under the hottest and driest conditions. These results demonstrated that not only is the biosynthetic pathway of phenolic monoterpenes in *T. capitata* favoured in high-temperature environments, as reported in other Lamiaceae, but also that carvacrol is present only in markedly “Mediterranean-like” environments [35]. In Spain, *T. capitata* EOs from wild and cultivated plants were studied, and all of them were classified as carvacrol chemotype [36]. Furthermore, *T. capitata* EOs from Portugal and from Turkey were found to be carvacrol chemotype [37,38]. The carvacrol chemotype is dominant in the majority of the studied *T. capitata* populations, being carvacrol the main compound in the EO from these populations. 

Several studies have been conducted on the allelopathic potential of *T. capitata*. In Israel, the suppression of several annuals, such as *Plantago psyllium* L. and *Erucaria hispanica* (L.) Druce, was observed around *T. capitata* formations. These effects were verified in the laboratory, as the germination of both species was inhibited by volatiles from *T. capitata* sprouts, as well as by their aqueous extracts and EOs. An autoallelopathic effect was also observed [39]. *T. capitata* EOs have shown phytotoxic effects on seed germination and seedling growth of various species [40,41,42]. *Sinapis arvensis* L. seeds soaked for 30 min in a solution of *T. capitata* EO (83.86% carvacrol) at 1.5 μL/mL with Tween 20 (0.1%), and placed in *Petri* dishes, did not germinate after 10 days of watering [43]. *T. capitata* EO (carvacrol 69.15%) also completely inhibited *S. arvensis* germination and strongly reduced that of *Phalaris canariensis* L. and *Lolium rigidum* Gaudin at 1 μL/mL in in vitro experiments [44]. Our previous research demonstrated that *T. capitata* EO has strong herbicidal activity against *Erigeron bonariensis* L., an important weed in many crops around the world which has developed resistance to many herbicides, including glyphosate [45].

Research on natural products as bioherbicides has greatly increased over the past few years, due to the shift in agricultural techniques to control weeds towards more sustainable ones, promoting integrated weed management (IWM) [46]. IWM is the combination of different methods for weed control: cultural, physical, mechanical, biological, biotechnological and chemical, giving priority to non-chemical ones whenever possible. The European legislation (Directive 2009/128/EC) supports the sustainable use of pesticides in the European Union. The overuse of synthetic herbicides caused negative effects in the environment and non-target organisms [47], and also promoted the development of herbicide-resistant weeds [48].

Bioherbicides are products of natural origin for weed control [49]. According to EPA (United States Environmental Protection Agency) biopesticides can be classified in three categories: (1) biochemical pesticides, which are natural substances that control pests by non-toxic mechanisms; (2) microbial pesticides, in which a microorganism is the active ingredient; and (3) plant-incorporated protectants, which are pesticide substances produced by plants from genetic material added to the plant [50]. The use of bioherbicides as tools for IWM allows many advantages, such as increased target specificity, rapid degradation, and less restrictive (sometimes non-existent) maximum residue limits [46,51]. In the context of IWM strategies, a properly managed application of bioherbicides can decrease the total need for synthetic herbicides [51].

The research carried out on the herbicidal activity of *T. capitata* has been mainly performed under in vitro conditions [42,43]. Our group has been investigating the herbicidal activity of *T. capitata* EO for over ten years, and this paper reports the results of in vitro and in vivo experiments. Since the biological activities of *T. capitata* EO were demonstrated to be due to its major compound, carvacrol [4,52,53], we have also tested carvacrol through in vitro and in vivo experiments, under greenhouse conditions. Finally, for a better understanding of the phytotoxic potential of *T. capitata* EO, we tested carvacrol on *Arabidopsis thaliana* (L.) Heynh. A dose–response curve for the germination and growth of *A. thaliana* was drawn, and the morphology of carvacrol-treated radicles was investigated.

Our studies demonstrated that *T. capitata* EO, and its main compound carvacrol, in either in vitro and in vivo conditions, exhibited great potential against many noxious Mediterranean weeds of cosmopolite distribution, and could be excellent candidates for bioherbicide formulations, which are a very important tool in the context of IWM, more respectful towards the environment and the ecosystems.

## 2. Results and Discussion

### 2.1. Thymbra Capitata EOs Composition

All the tested EOs were carvacrol chemotype (Table 1). This chemotype is dominant in most studies about *T. capitata* EO and its biological activities [36,42,54,55], although thymol and thymol/carvacrol chemotypes have been described [17,55,56]. The samples obtained from *T. capitata* populations from Sicily (TC1 and TC2) contained as main compounds carvacrol, *p*-cymene, and β-caryophyllene (ranked in decreasing order of abundance) (Table 1). Carvacrol content was higher in the EO obtained from plants at flowering stage (TC1, 77.02%) than at vegetative stage (TC2, 65.55%). *T. capitata* biotypes from Sicily have been characterized and resulted belonging to the carvacrol chemotype [54]. The main compounds in the samples obtained from *T. capitata* growing in Spain were carvacrol, *p*-cymene and γ-terpinene (Table 1). Other studies of *T. capitata* populations from Spain also found that all the samples studied, from wild and cultivated populations were carvacrol chemotype [36]. The high content in carvacrol is associated to markedly “Mediterranean-like” environments [35].

### 2.2. In Vitro Herbicidal Activity Experiments

#### 2.2.1. Herbicidal Activity of TC1, TC2 and Carvacrol against *Portulaca oleracea* and *Erigeron canadensis*

Both EOs tested, TC1 and TC2, showed similar effects on *P. oleracea* and *E. canadensis* seed germination (Table 2). *E. canadensis* was more sensitive than *P. oleracea* to *T. capitata* EO, as all treated seeds did not germinate at all assayed concentrations. The application of *T. capitata* EO inhibited seed germination of *P*. *oleracea*. The lowest concentration tested (0.125 μL/mL) reduced germination by 47.1% (TC1) and by 55.2% (TC2) when compared to the control. There were no significant differences in the phytotoxic effects caused on *P. oleracea* seeds between the other tested concentrations, reducing *P. oleracea* germination to 0 or values close to 0 (Table 2. Carvacrol inhibited completely seed germination on both species at all applied doses (Table 2).

**Figure 1:**
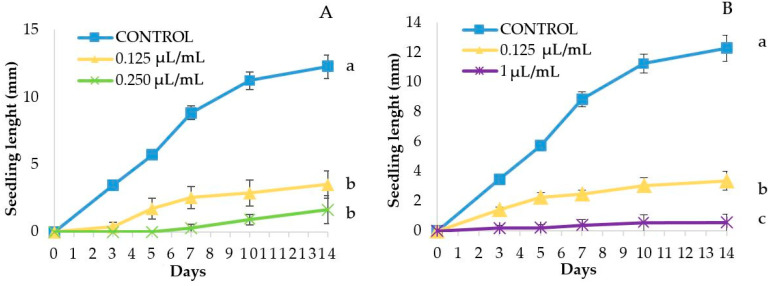
Seedling length (mm) (mean ± SE) in *Portulaca oleracea* control or treated with TC1 (**A**) and TC2 (**B**). Different letters at the end of the growth curves indicate significant differences among doses (*p* < 0.05) using Fisher’s least significant difference (LSD) test.

Regarding the phytotoxic effects on seedlings, both EOs reduced significantly the treated seedling length at all tested concentrations (Figure 1). The seedlings were strongly affected, also at the lowest dose (0.125 μL/mL), being 71.4% (TC1, Figure 1A) and 72.6% (TC2, Figure 1B) shorter than the controls. The seedlings treated at the highest dose (1 μL/mL) exhibited a significantly reduced length as compared to the lowest dose (0.125 μL/mL), and were 95.4% shorter than the controls (Figure 1B).

**Figure 2:**
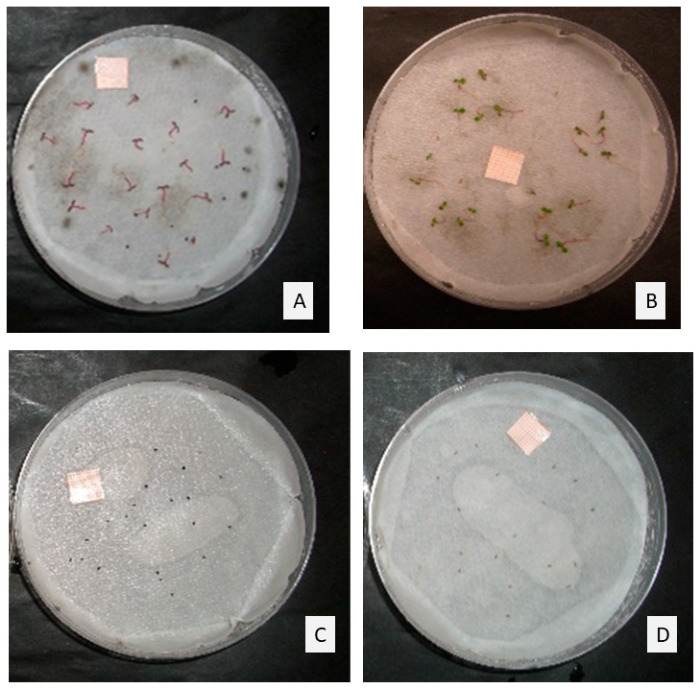
Seeds in control plates of (**A**) *Portulaca oleracea*, and (**B**) *Erigeron canadensis*, and seeds treated with the maximum doses of TC1 (1 µL/mL) of (**C**) *P. oleracea*, and (**D**) *E. canadensis*, after 14-days incubation.

In Figure 2, *E. canadensis* and *P. oleracea* plates control and treated with the maximum doses of TC1 (1 μL/mL) at the end of the experiment can be observed. The EO completely blocked the germination of both species.

#### 2.2.2. Herbicidal Activity of TC3 against *Solanum nigrum*, *Chenopodium album*, *Sonchus oleraceus* and *Setaria verticillata*

The most sensitive species to TC3 EO were *C. album* and *S. oleraceus*, as their germination was blocked at all the applied doses (Table 3). The germination of *S. nigrum* and *S. verticillata* was completely inhibited after seeds treatment at the two highest concentrations of TC3, 0.5 and 1 μL/mL (Table 3). The lowest doses of the EO tested (0.125 and 0.25 μL/mL) also showed strong phytotoxic effects, inhibiting by 73.7% and by 75.8% *S. nigrum* germination and by 80% and by 86.3% *S. verticillata* germination, respectively.

**Figure 3:**
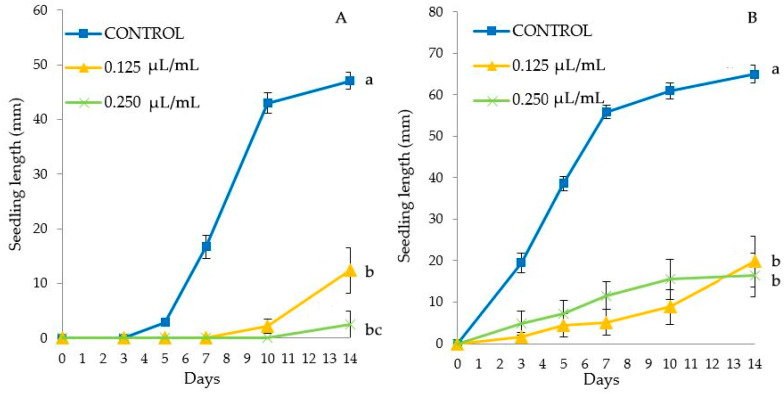
Phytotoxic effects of *Thymbra capitata* EO (TC3) on *Solanum nigrum* (**A**) and *Setaria verticillata* (**B**) seedling length (mm) (mean ± SE), measured for 14 days. Different letters at the end of the growth curves indicate significant differences among doses (*p* < 0.05) using Fisher’s least significant difference (LSD) test.

In *C. album* and *S. oleraceus*, seedling length could not be evaluated, because no seed germinated after the treatment at the tested EO concentrations (Table 3 and Table 4). The same happened at the highest concentrations (0.5 and 1 μL/mL) in *S. nigrum* and *S. verticillata* (Table 3 and Table 4). The lowest doses of TC3 (0.125 and 0.25 μL/mL) significantly reduced the length of the treated seedlings as compared to the control: for each concentration, by 73.66% and by 94.77% of seedling length reduction was measured in *S. nigrum* (Figure 3A), and by 69.54% and by 74,54% in *S. verticillata*, respectively (Figure 3B). 

#### 2.2.3. Herbicidal Activity of TC4 against *Amaranthus retroflexus*, *Portulaca oleracea*, *Avena fatua* and *Echinochloa crus-galli*

*E. crus-galli* and *P. oleracea* behaved as the most resistant species to TC4 EO, since only the highest dose (2 μL/mL) prevented their germination (Table 5). Nevertheless, the germination of all species was lower as the doses of the EO increased (Table 5). The germination of the other tested species was inhibited completely at doses of 0.5 μL/mL for *A. fatua*, which was the most sensitive species, and 1 μL/mL for *A. retroflexus* (Table 5). All treatments showed significant differences when compared to the control, except for the lower dose applied to *A. fatua*. (Table 5).

**Figure 4:**
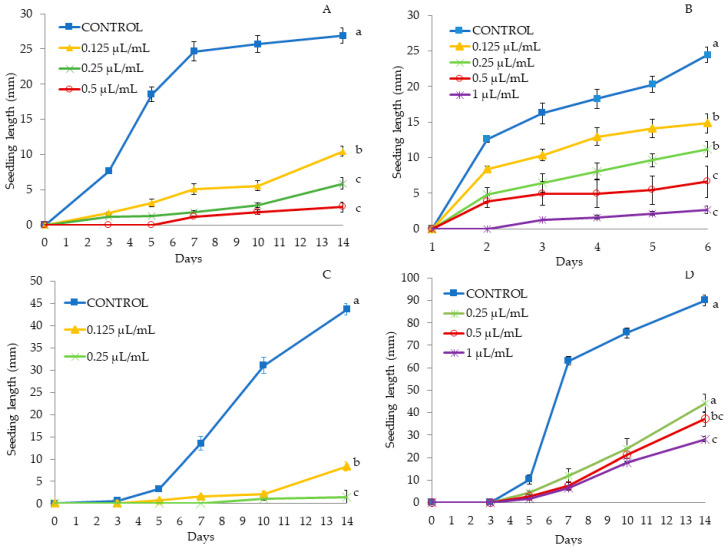
Phytotoxic effects of *Thymbra capitata* EO (TC4) on *A. retroflexus* (**A**), *P. oleracea* (**B**), *A. fatua* (**C**) and *E. crus-galli* (**D**) seedling length (mm) (mean ± SE), measured for 14 days. Different letters at the end of the growth curves indicate significant differences among doses (*p* < 0.05) using Fisher’s least significant difference (LSD) test.

Figure 4 shows the results of seedling growth over time in the different species. All treated seedlings presented a significant reduced length as compared to control, except *E. crus-galli* seedlings treated at the lowest TC4 dose (0.25 µL/mL, as the 0.125 dose was not tested on this species). The maximum reduction of seedling length was found for the highest applied dose, being by 96.56% for *A. fatua* (with 0.25 µL/mL dose) (Figure 4C), by 90.56% for *A. retroflexus* (0.5 µL/mL) (Figure 4A), by 89.00% for *P. oleracea* (1 µL/mL) (Figure 4B) and by 68.70% for *E. crus-galli* (1 µL/mL) (Figure 4D). 

Finally, in seedlings of *A. fatua* and *E. crus-galli* the coleoptile and radicle length were separately measured (Table 6), with the purpose to determine if any phytotoxic effect could be observed, specifically addressed to one of these parts. The results showed a similar length reduction on both radicle and coleoptile for the two species, being *E. crus-galli* more resistant to TC4 than *A. fatua* (Table 6). The maximum observed reductions were 95.07% and 98.15% for *A. fatua* coleoptile and radicle (0.250 µL/mL dose), and 69.77% and 67.28% for *E. crus-galli* coleoptile and radicle, respectively (Table 6).

The performed in vitro experiments allowed concluding that *T. capitata* EO showed a broad-spectrum herbicidal activity, being able to control the germination and seedling growth of all tested weeds. However, each species exhibited different sensitivity to *T. capitata* EO, from the most sensitive to the more resistant ranking as follows: *E. canadensis*, *S. oleraceus* and *C. album* were the most sensitive, as their germination was blocked at all tested concentrations; *S. verticillata*, *A. fatua* and *S. nigrum* were intermediate, as their germination was completely inhibited at concentrations equal to or higher than 0.5 µL/mL; *A. retroflexus* germination was prevented at concentrations 1 and 2 µL/mL; *P. oleracea* and *E. crus-galli* were the most resistant, being their germination completely controlled at the highest tested dose, 2 µL/mL. The doses of *T. capitata* EO that did not prevent weed germination caused an abnormal development of seedlings, along with lower growth rates.

Some experiments investigated the herbicidal activity of *T. capitata* EO and its main compound carvacrol, in in vitro conditions, and all corroborated the excellent herbicidal potential of this EO and its main compound [42,43,57]. The herbicidal potential of *T. capitata* EO from Tunisia with 69.15% carvacrol was studied in in vitro assays against *S. arvensis*, *P. canariensis* and *L. rigidum* at concentrations of 0.25, 0.5, 0.75 and 1 µL/mL. At the highest tested dose, *T. capitata* EO completely controlled the germination of *S. arvensis*, and reduced the germination of *P. canariensis* and *L. rigidum* by 82.1% and by 92%, respectively [43]. In this research, similar concentrations of *T. capitata* EO were tested, and also in this case weed species showed a different sensitivity to *T. capitata* EO, which supports our results. In addition, the authors verified a loss of vigor of the treated weeds. Similarly, other EOs rich in carvacrol showed a good herbicidal potential [58,59]; for example, *Satureja hortensis* L. EO, with 55.6% carvacrol, inhibited *A. retroflexus* and *C. album* germination [58]; *Plectranthus amboinicus* (Lour.) Spreng. EO, with 88.6% carvacrol, slowed and inhibited *Lactuca sativa* L. and *Sorghum bicolor* (L.) Moench germination, also causing a decrease in their growth [58,59].

### 2.3. In Vivo Herbicidal Activity Experiments

#### 2.3.1. Pre-Emergence Assays with TC3 against Sown Target Seeds, and Weeds Contained in the Soil Seedbank in Pots under Greenhouse Conditions

Three application methods were compared for TC3 supplying to the soil, using the same concentrations (1, 2 and 4 µL/mL) to find the most effective system to control weeds maximizing the EO phytotoxic effects: application of TC3 directly injected to the soil (I1, I2 and I4), application of TC3 emulsified with Tween 20 (T1, T2 and T4), and application of TC3 emulsified with Fitoil (F1, F2 and F4).

##### Phytotoxic Activity of TC3 Injected Directly into the Soil

**Figure 5:**
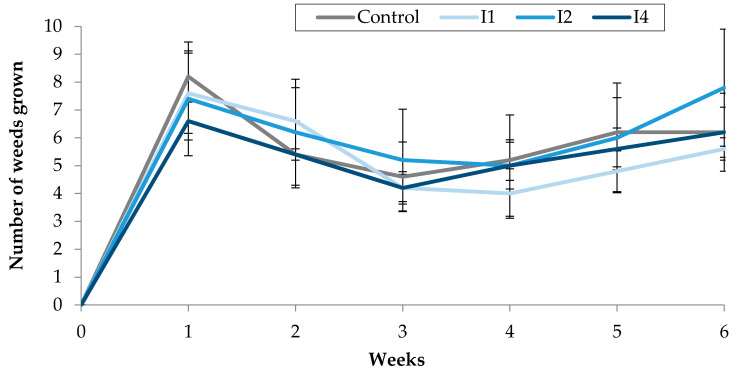
Effect of TC3, injected into the soil, on the number of weeds grown in treated pots.

After the direct injection into the soil of the three different concentrations of TC3 applied (1 (I1), 2 (I2) and 4 (I4) µL/mL), no significant difference was observed between the number of weeds growing in the treated pots, and those counted in the control pots. A comparable number of plants emerged from the soil in the different treatments throughout the 6-weeks long experiment (Figure 5). 

##### Phytotoxic Activity of TC3 Supplied with Water, Using Tween 20 as Emulsifier and Applied by Irrigation to the Pots

**Figure 6:**
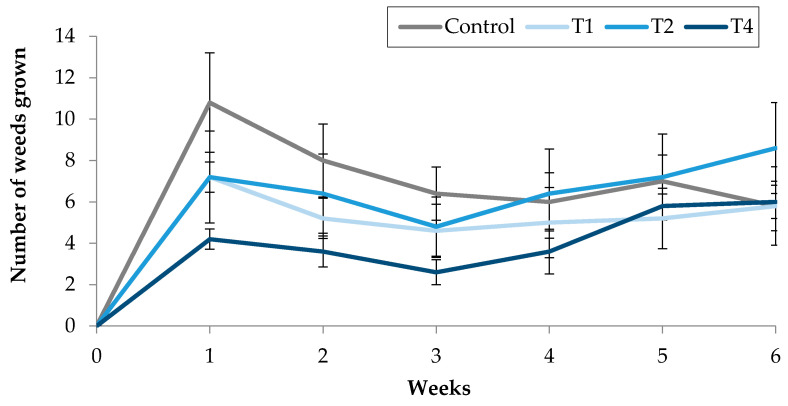
Effect of TC3, supplied with water, using Tween 20 as emulsifier, on the number of weeds grown in treated pots.

The first week after treatments application, statistically significant differences were observed between the number of weeds grown in the control pots and those counted in the treated pots, with a 61% reduction in the number of weeds grown in the pots treated with the highest EO concentration (4 µL/mL, T4) (Figure 6). Oppositely, no difference was appreciated between the numbers of plants after the application of the three EO concentrations. For the following weeks, although no statistically significant difference was detected between the control and the remaining treatments, the number of weeds grown in the pots treated at the highest concentration (4 µL/mL) was lower than those in the control pots, being the two values overlapping in the fifth and sixth weeks (Figure 6).

##### Phytotoxic Activity of TC3 Supplied with Water, Using Fitoil as Emulsifier and Applied by Irrigation to the Pots

**Figure 7:**
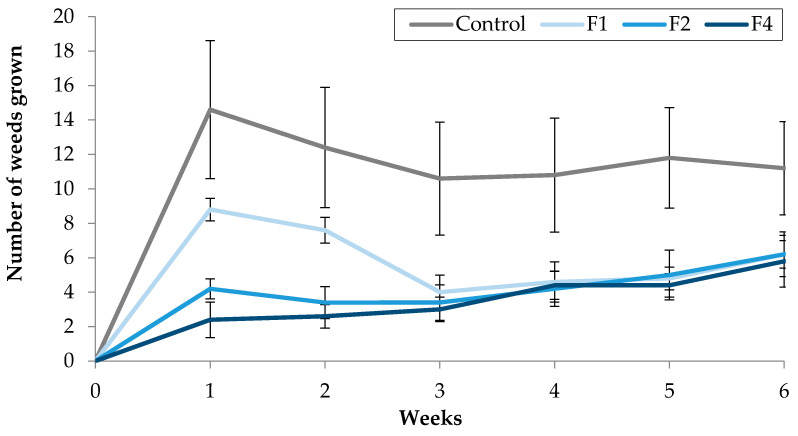
Effect of TC3, supplied with water, using Fitoil as emulsifier, on the number of weeds grown in treated pots.

During the 6-weeks trial, the number of weeds grown in the control pots was higher than in the pots treated with the three concentrations of TC3 emulsified with Fitoil (F1, F2 and F4) (Figure 7), being these differences statistically significant. The strongest phytotoxic effects were observed the first two weeks, with an 83.56% reduction of the number of weeds in pots treated with F4, compared to the control (Figure 7). In the same weeks, significant differences were also observed between the number of weeds grown in the pots treated with F1, F2 and F4, but from week 3 until the end of the experiment, all treated pots presented a similar number of weeds, lower than control, achieving at the end of the trial a 40% reduction in the number of weeds grown per pot compared to control. 

From these results, we can conclude that the most effective application method was supplying the EO emulsified with water, using Fitoil as emulsifier, by irrigation to the pots. In this case, the phytotoxic activity was maintained for the whole six weeks experiment. Contrastingly, EO supplied with water and emulsified with Tween 20, only maintained its activity for two weeks, and the injected EO was not effective at any time. The highest concentrations were the most effective during the first weeks, in the treatments with EO emulsified with Tween 20 and Fitoil, although in the case of EO emulsified with Tween 20 no statistically significant differences could be appreciated. In contrast, the activity of the three applied concentrations was similar after the third week. 

In a recent greenhouse study with *T. capitata* EO injected into a soil obtained from an organically managed citrus orchard, in which weed seeds were not added to the soil, the concentration of 2 μL/mL lowered the number of emerging plants up to 74.1% compared to the control [60]. However, in the present work this method did not show such good results. This difference was probably due to factors such as the different seedbank composition of each soil, since the EO activity depends on the treated species, or the environmental conditions within the greenhouse. A fluctuation in the greenhouse relative humidity, possibly due to a failure in the greenhouse cooling system, resulting in a moisture decrease from the second to the third week, could affect seedling development, mainly in the control pots as they held the first seeds to emerge, diminishing the differences between treated and control pots.

Further research is necessary about the formulations and mode of application of *T. capitata* EO, as they can deeply affect EO activity, especially in in vivo conditions.

#### 2.3.2. Pre-Emergence Assays with TC4 against the Spontaneous Weeds Contained in the Soil Seedbank

In this assay, TC4 was tested at doses of 1, 2 and 4 μL/mL, applied at different volumes (5.5, 2.775 and 1.83 L/m^2^), in order to find the best combination for achieving the maximum herbicidal EO effect. 

**Figure 8:**
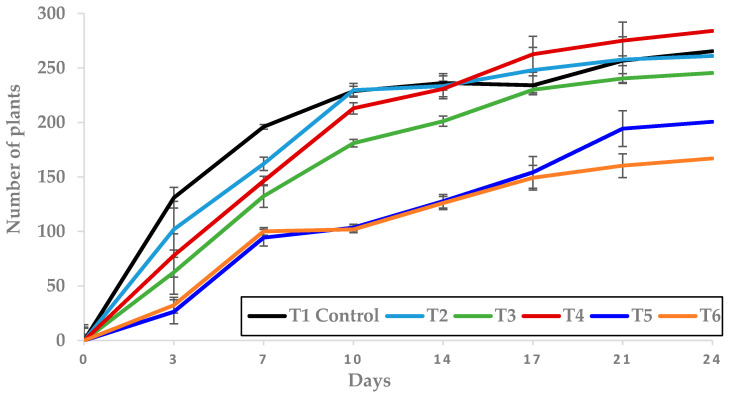
Effect of T1 to T6 treatments applied in pre-emergence on the number of plants grown (mean ± standard error) in the trays where they were applied.

The strongest herbicidal effects were observed on the third day, being T5 and T6 the most phytotoxic treatments, as the number of plants was reduced by 80.1% and by 72.6%, respectively, when compared to the control. In contrast, both treatments showed 24.2% and 37% fewer plants than the control, respectively, when counting was made after 24 days (Figure 8). These results suggest the possibility to obtain the same herbicidal effect with a smaller quantity of EO, when applied at certain doses. This is very important, as it involves significant savings in terms of the amount of EO to apply. A larger number of tests is necessary, at different doses and volumes, not just under greenhouse conditions, but also in the field, in order to determine optimal doses and volumes for weed control that can be economically competitive with the synthetic herbicides that are already on the market. The results obtained using different TC4 concentrations corroborate the outcomes from other tests previously carried out by the IAM natural herbicide research group [53,60,61], which also showed that the differences between treatments decrease throughout the test run, probably due to the low persistence of the EO.

#### 2.3.3. Post-Emergence Assays with TC4 against the Spontaneous Weeds Emerged from The Soil Seedbank

**Figure 9:**
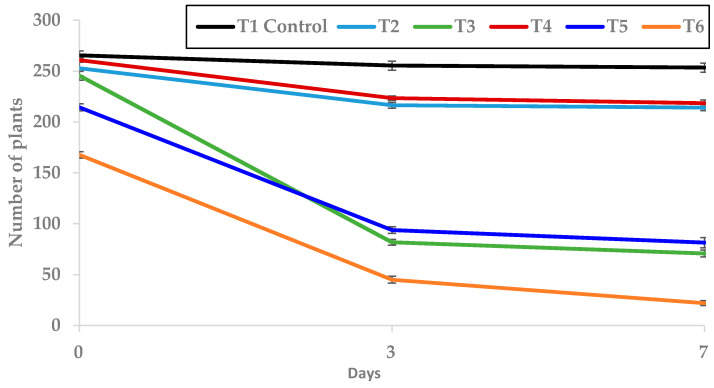
Effect of T1 to T6 treatments applied in post-emergence on the number of plants grown (mean ± standard error) in the trays where they were applied.

The same treatments tested in pre-emergence assays were applied in post-emergence, in the same trays. Significant differences were observed in the number of plants of the control trays when compared to all the EO treated trays (Figure 9). Significant differences between treatments were also found, the most effective being T6, which presented a number of plants 82.4% lower than the control, followed by T3 and T5, with a decrease of 67.9% and 63.1%, respectively, on the third day after treatment application (Figure 9). These differences were maintained for 7 days, being the number of grown plants significantly reduced compared to the control by 91.3% (T6), by 71.9% (T3), by 68% (T5), by 15.4% (T2) and by 13.9% (T4). Differences between T2 and T4, and T3 and T5 were not significant (Figure 9).

The obtained results offered another alternative for supplying the EOs, as pre-emergence application gave better results in in vitro conditions than post-emergence treatments [53]. Under greenhouse conditions, EOs are more exposed to volatilization than in in vitro tests, and for this reason, pre-emergence application of the EO could result in higher dispersion without herbicidal activity than post-emergence application, in which the EO comes in contact with the plant immediately, producing its phytotoxic effects. Additional tests are necessary, both in greenhouse and in field conditions, in order to establish doses and protocols regarding the ideal application times to achieve optimal control of weeds with *T. capitata* EO. Several studies have obtained good results in weed control using other EOs, such as clove EO [62], *S. hortensis* EO [58] or EOs from Asteraceae species applied in post-emergence [63].

#### 2.3.4. Post-Emergence Assays with TC4 against Target Weeds *Portulaca oleracea*, *Avena fatua* and *Echinochloa crus-galli* Applied by Spraying

The most sensitive species to TC4 applied by spraying was *P. oleracea*, followed by *A. fatua* and *E. crus-galli*, with efficacies of 60, 32 and 0, respectively (Table 7). TC4 was more effective when applied at higher doses (Table 7).

In Table 8, Table 9 and Table 10 are reported the phytotoxic effects of TC4 on the weed species tested. These tables can be found including the standard error in supplementary materials (Tables S8–S10). On *P. oleracea*, all the doses tested of TC4 presented the same efficacy (100), producing the highest damage level (3). All *P. oleracea* plants treated with TC4 died.

The lowest dose of TC4 applied (4 µL/mL) was not effective to control *A. fatua* plants (efficacy 0) but it caused a damage level near 2, and reduced significantly the length of aerial parts and roots (Table 9). The phytotoxic effects were more severe as higher doses were applied. Only the maximum dose controlled all plants (efficacy 100), however, the medium dose induced a high damage level, significantly reducing all the measured biometric variables (Table 9). Is important to consider not only the efficacy but also the damage level because damaged plants, in field conditions would be less competitive with crops for nutrients and natural resources.

TC4 at the tested doses did not control *E. crus-galli* plants (0 efficacy, Table 10). The highest applied dose, reduced significantly all the biometric variables and caused a damage level of 1.90, which is a medium damage, not severe (Table 10). 

An herbicide is effective when it reaches its site of action. To achieve this, the herbicide must cross the cuticle, which is an extracellular lipid layer with a 0.5–15 µm thickness, generally composed of cutin and embedded waxes, with epicuticular waxes on the outer surface. While cutin components leave spaces that can be crossed, the cuticular waxes represent the main barrier to the diffusion of lipophilic compounds because of their physical structure [64]. The different sensitivity of the studied species to *T. capitata* EO when supplied by spraying could be explained by the structural differences in their leaf cuticle as concerns waxes and cutin content, since these components are responsible for the absorption properties of each species [65]. The leaf stage has a preeminent role in diffusion mechanism, because as plants grow up there is an increase in the deposition of different chemical cuticular compounds, ultimately hampering the herbicide absorption [66]. 

The cuticle of *P. oleracea* was studied and it showed an undulated surface with peaks and valleys, with stomata located in depressions. When observed by means of transmission electron microscopy (TEM), the cuticle transections showed a rather thin continuous cuticle, 0.2 to 0.5 µm thick. Epicuticular waxes were not observed with scanning electron microscopy (SEM) or TEM [64]. This could probably explain the easier absorption of *T. capitata* EO in *P. oleracea* than in *A. fatua* or *E. crus-galli*. In laboratory observations, the leaf surface and epicuticular wax content of *E. crus-galli* leaves were examined. Stomata and trichomes were present on adaxial and abaxial leaf surfaces, being stomata and trichomes more abundant on the abaxial than on the adaxial leaf surface. The mean value of the wax content per leaf area unit was 35.9 μg/cm^2^ [67]. Epicuticular wax deposition was measured in *A. fatua* leaves being 34 µg/cm^2^. More than 90% of the epicuticular wax was constituted by the primary alcohol 1-hexacosanol [67].

The surfactant used to optimize herbicidal activity is very important as well [68]. Further studies should be carried out to find out which surfactants could enhance the herbicidal activity of *T. capitata* EO when applied by spraying.

In Table 11 are reported efficacy results of this assay and of our previous works about herbicidal activity of *T. capitata* on target weed species to compare the efficacy when applying TC4 EO by irrigation or by spraying. Comparing the results of both supplying methods, can be observed that in monocotyledons (*A. fatua* and *E crus-galli*), the same applied EO doses are more effective when supplied by irrigation than by spraying. On the contrary, on dicotyledons (*P. oleracea* and *E. bonariensis*) the same doses were more effective when applied by spraying. In some species (*A. fatua*) the highest dose was equally effective, but at lower doses the differences in effectivity between the two application systems could be clearly appreciated.

The higher efficacy of watering-administered TC4 EO on monocots than on dicots could be related to the number and structure of xylem elements in these species. As known, monocotyledonous species often exhibit stems with scattered vascular bundles, and no pith and cortex are delineated [69]. Although the functional consequences of these distinct organization are not well understood at either the organ or the whole-organism level [70], the higher number of xylem elements in monocot species could be influencing the EO phytotoxic effects by increasing its translocation from the roots to the aerial parts.

On the other side, the higher efficacy of sprayed TC4 EO on dicots than on monocots could be related, as previously mentioned, to differences in the cuticle of these species, which could modify EO penetration into the leaves. Although some studies have not found significant correlation between cuticle thickness and penetration of compounds [71,72], others [73] have reported the major importance that ultrastructural cuticle features can have on the solubility of herbicides, which can partly explain the variability of cuticular permeability among species [74,75]. The differences in cuticle ultrastructure among dicot and monocot species could therefore explain the stronger phytotoxic effects observed on dicot species *P. oleracea* and *E. bonariensis* when are sprayed with TC4 EO. 

When considering the herbicidal activity of EOs, many aspects must be taken into consideration, not only concerning the active doses, but also the way in which EOs are formulated and applied, because both factors can influence the EO activities. Another key point is the optimum phenological stage for EO application to weeds and crops, with the goal to cause maximum damage on weeds and no damage in crops. The different efficacy of TC4 EO sprayed on monocots and dicots, could be exploited for weed management in monocot crops, by spraying dicots, taking into account the safe stage of development for the crop not to be damaged. 

A better understanding of the mechanism of action of *T. capitata* EO, however, would be necessary to optimize its formulations and administration methods, in order to maximize its herbicidal effects. 

#### 2.3.5. Post-Emergence Assays with TC4 and Carvacrol against *Avena fatua*, Applied by Irrigation and Spraying

In this assay, TC4 and its main compound carvacrol were tested by irrigation and spraying on *A. fatua*, a sensitive species to *T. capitata* EO, to compare their phytotoxic effects. The most effective treatment to control *A. fatua* was TC4 at 8 µL/mL applied by irrigation, as it killed all the plants (100 efficacy) (Table 12). Both, the EO and carvacrol were more effective applied by irrigation than by spraying (Table 12). When applied by irrigation, at the low dose tested (4 µL/mL) TC4 EO was significantly less effective than carvacrol, which did not show significant differences between doses (Table 12). At the highest dose tested (8 µL/mL) there were no significant differences on efficacy between carvacrol and TC4 EO, although the EO achieved 100 efficacy and carvacrol 90. When applied by spraying, no significant differences were found in efficacy between carvacrol and TC4 EO, at both doses tested. In this case, the EO showed highest efficacy values than the singular compound, although the differences were not significant (Table 12); however, the EO showed greater phytotoxic effects in the biometric variables measured than carvacrol (Table 12). It could be hypothesized that any compound present in the EO helped carvacrol to penetrate better through the leaves. It has been demonstrated that synergistic interactions between major and minor compounds present in the EOs can influence several characteristics of EOs, such as hypo- or hydrophilic interaction, increasing the solubility of compounds and cuticular penetration [76]. More studies are necessary to understand better the differences discovered in efficacy between the supplying methods and between the EO and carvacrol. These findings must be taken in to consideration for achieving the maximum herbicidal activity of EO and carvacrol.

In supplementary materials can be found Table 12 including standard error (Table S12).

#### 2.3.6. Phytotoxic Activity of Carvacrol in *Arabidopsis thaliana*

**Figure 10:**
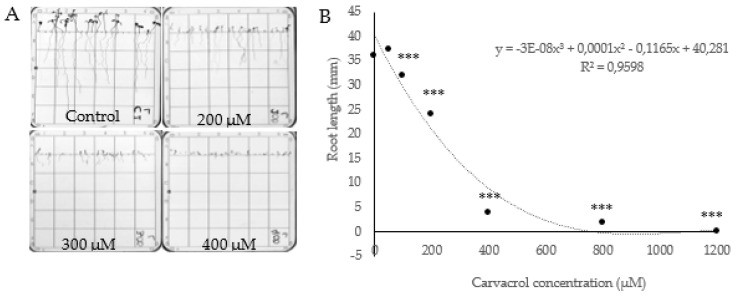
(**A**) Scanned images of *Arabidopsis thaliana* seedlings treated with 0, 200, 300 and 400 μM carvacrol (**B**) Dose–response curve of radicle length of *Arabidopsis thaliana* seedlings after 14 d of carvacrol treatment (0, 100, 200, 400, 800, 1200 μM). Points marked with asterisks are significantly different from the control (*p* ≤ 0.05).

Carvacrol treatment did not significantly affect *A. thaliana* germination. However, the root growth and shoot development of carvacrol-treated *A. thaliana* seedlings was strongly affected by this compound (Figure 10). Roots were shorter and thinner after carvacrol treatment, with a particularly strong inhibition between 200 and 400 μM (Figure 10A). Carvacrol-treated roots were characterized by a zig-zag development, and at higher concentrations (400 μM), roots growing in different directions could be observed (Figure 10A). Moreover, shoot development was already inhibited at low concentrations and development of true leaves was not observed in carvacrol-treated seedlings. The range of tested concentrations (0–1200 μM) included the LCIC (Low Complete Inhibiton Concentration, the concentration at which the inhibition of growth is complete) as can be seen in Figure 10B, where 1200 μM-treated seeds showed no growth of roots or shoots. 

**Figure 11:**
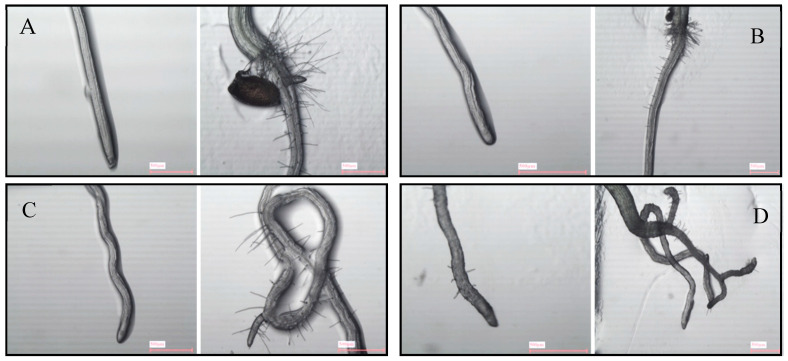
Apical region and transition zone of *A. thaliana* roots after 14 days of growth in agar with 0 (**A**), 100 (**B**), 200 (**C**), and 400 (**D**) μM carvacrol. Images were taken with a magnifier (Nikon SMZ 1500, Melville, NY, USA).

Magnifier analyses of carvacrol-treated roots (Figure 11) revealed a clear torsion in radicles of *A. thaliana* treated with carvacrol, already at low concentrations (100 and 200 µM). While control roots showed symmetric cell rows growing straight according to gravitropism (Figure 11A), carvacrol-treated roots showed disorganized rows of cells (Figure 11B), that started to grow without a pattern when carvacrol concentrations were higher (Figure 11C), even losing the gravitropic perception at the tested stronger concentrations (Figure 11D). This torsion can be detected as a zig-zag growth of the root (Figure 11C) but also as a spiral growth of primary and secondary roots (Figure 11B,C). As already known [77,78,79], torsion effects are related to the loss of gravitropism that is usually associated with alterations in the organization of the microtubules, resulting in the inhibition of root elongation [80]. 

Moreover, carvacrol reduced the number and length of the root hairs at the transition zone between hypocotyl and epicotyl (Figure 11), but increased the presence of ectopic roots close to the root tip, which have been previously related to auxin unbalance and altered microtubule disposition [77,81,82].

## 3. Materials and Methods 

### 3.1. *Thymbra Capitata* EOs and Carvacrol Used for the Experiments

#### 3.1.1. *Thymbra capitata* EOs Obtained by Hydrodistillation from Collected Plant Material (TC1, TC2 and TC3)

*Thymbra capitata* EO 1 (TC1)—Aerial parts from *Thymbra capitata* (L.) Cav. were collected in June 2006, at full bloom, from Enna (Enna province, Sicily, Italy) 

*T. capitata* EO 2 (TC2)—Aerial parts from *T. capitata* were collected in January 2010, at vegetative stage near Riesi (Caltanissetta province, Sicily, Italy).

*T. capitata* EO 3 (TC3)—Aerial parts from *T. capitata* were collected from the surroundings of Carmona (Seville province, Spain) in July 2012.

Voucher specimens of all collected samples were deposited in the herbarium of the Universitat Politècnica de València (VALA 9486, 9487 and 9488). *T. capitata* EOs from plants collected in Italy (TC1 and TC2) were obtained by steam distillation (hydrodistillation) using an Albrigi Luigi EO extractor of 20 L (Albrigi Luigi SRL, Verona, Italy). The bottom of the extractor was filled with tap water, that did not exceed the level established by a grate previously settled at the bottom. Then, the fresh plant material was introduced in the distiller and a top grill was placed above it. Once the extractor was closed hermetically, it was heated by means of a flame generated by butane gas, producing steam, which transported the volatile components liberated from the raw material. The vapor was condensed as they passed through the cooler, collecting the EO in a burette. This process was maintained for at least 3 h, finishing when no additional EO was extracted for 30 min. The average yield expressed in v/w (volume of EO obtained in milliliters, per grams of distilled plant) of *T. capitata* was 2.49% at bloom stage and 0.10% at vegetative stage. 

TC3 was obtained by hydrodistillation using a Clevenger-type apparatus. The fresh plant material was introduced in round-bottom flasks of 2 and 4 L and distilled water was added (1000 mL in the 2 L flask or 2000 mL in the 4 L flask). Heat was applied to the round-bottom flasks by heating mantles in order to generate water vapor, carrying the volatile compounds of the drug. Then, it was cooled in the condenser and passed to the graduated collector tube, where the EO was separated from the water. This process was carried out for at least 3 h, until no additional EO was extracted for 30 min. TC4 yield was 3%. All the obtained EOs were stored at 4 °C, until they were analyzed or tested.

#### 3.1.2. *Thymbra capitata* EO and Carvacrol Purchased (TC4)

*T. capitata* EO 4 (TC4) was purchased from Bordas S.A. (Seville, Spain). 

Carvacrol was purchased from Sigma-Aldrich (St Louis, MO, USA).

#### 3.1.3. EOs Composition. GC and GC-MS Analyses

The EOs constituents were quantified by gas chromatography using a Clarus 500GC Perkin–Elmer apparatus equipped with a flame ionization detector (FID), and a capillary column ZB-5 (30 m × 0.25 mm i.d. × 0.25 μm film thickness). The injection volume was 1 μL. The GC oven temperature was set at 60 °C for 5 min, with 3 °C increases per min to 180 °C, then 20 °C increases per min to 280 °C, which was maintained for 10 min. Helium was the carrier gas (1.2 mL/min). Injector and detector temperatures were set at 250 °C. The percentage composition of the EO was computed from GC peak areas without correction factors by means of the software Total Chrom 6.2 (Perkin-Elmer Inc., Wellesley, PA, USA).

For the identification of the compounds, gas chromatography coupled to mass spectrometry (GC–MS) was performed using a Clarus 500 GC–MS from Perkin-Elmer Inc. apparatus equipped with the same capillary column, carrier, and operating conditions as described for GC analysis. The ionization source temperature was set at 200 °C and an electron impact mode of 70 eV was employed. MS spectra were obtained by means of total ion scan (TIC) mode (mass range m/z 45–500 uma). The total ion chromatograms and mass spectra were processed with Turbomass 5.4 software (Perkin-Elmer Inc., Waltham, MA, USA). Retention indexes were determined by injection of C8–C32 n-alkanes standard under the same conditions. The EO components were identified by comparison of their mass spectra with those of the computer library NIST MS Search 2.0 and available data in the literature [83]. 

### 3.2. In Vitro Phytotoxic Assays

#### 3.2.1. Weed Seeds

The weeds selected for the experiments are important weeds in Mediterranean crops, with a cosmopolite distribution, many of which have developed herbicide-resistant biotypes [84].

##### Experiment 1 (2009)

*Portulaca oleracea* L. mature plants were collected from Vall d’Alba fields (Castellón province, Spain) in July 2007. *Erigeron canadensis* L. mature plants were collected from Bagheria fields (Palermo province, Sicily, Italy) in October 2009. The weeds were dried for 15 days at room temperature, afterwards the seeds were extracted. Uniform mature healthy seeds were selected and stored at room temperature until germination tests were performed.

##### Experiment 2 (2012)

*Setaria verticillata* (L.) P. Beauv. mature plants were collected from fields in the surroundings of Universitat Politècnica de València (Valencia province, Spain) in November 2011. The other weeds used in this experiment, were all collected in October 2012. Mature plants of *Solanum nigrum* L. were collected from Villena fields (Alicante province, Spain); *Chenopodium album* L. and *Sonchus oleraceus* (L.) L. mature plants were collected from Santo Domingo de la Calzada fields (Logroño province, Spain).

##### Experiment 3 (2018)

Seeds of *P. oleracea*, *Amaranthus retroflexus* L. and *Avena fatua* L. were purchased from Herbiseed (Reading, United Kingdom) in 2017, and seeds of *Echinochloa crus-galli* (L.) P. Beauv. were collected from rice fields in Sollana (Valencia province, Spain) in September 2017.

#### 3.2.2. Herbicidal Activity Assays in Petri Dishes

##### Experiment 1 (2009)—Effects of TC1, TC2 and Carvacrol on *Erigeron canadensis* and *Portulaca oleracea*

Sets of 20 seeds each, with five replicates per treatment, were placed in Petri dishes (9 cm diameter) between two layers of filter paper (50 g/m^2^) wetted with 4 mL of distilled water. TC1, TC2 and carvacrol, were added at volumes of 0, 0.5, 1, 2 and 4 µL to obtain concentrations 0 (control), 0.125, 0.25, 0.5 and 1 µL/mL. Petri dishes were sealed with Parafilm. According to previous assays, *E. canadensis* and *P. oleracea* seeds were incubated in a WTB incubator (Binder GmbH, Tuttlingen, Germany) at a constant temperature of 25.0 ± 0.1 °C, with a photoperiod of 12 h light and 12 h darkness. To evaluate the phytotoxic activity of the EOs and carvacrol, germination and seedling length were recorded after 3, 5, 7, 10 and 14 days. Images of the Petri dishes were registered and then processed by UTHSCSA Image Tool 3.0 (University of Texas Health Science Center, San Antonio, TX, USA) software.

##### Experiment 2 (2012)—Effects of TC3 on *Solanum nigrum*, *Chenopodium album*, *Sonchus oleraceaus* and *Setaria verticillata*

Petri dishes were prepared following the same methodology described above for Experiment 1, and incubated in an APG-GROW germination chamber (Climax, Barcelona, Spain) at 30.0 ± 0.1 °C for 16 h of light, and 20.0 ± 0.1 °C for 8 h of darkness. TC3 was tested at the same concentrations that TC1, TC2 and carvacrol (0.125, 0.25, 0.5 and 1 µL/mL). To assess the herbicidal activity of TC3 EO images from the Petri dishes were registered and processed as indicated above for Experiment 1. 

##### Experiment 3 (2018)—Effects of TC4 on *Amaranthus retroflexus*, *Portulaca oleracea*, *Avena fatua* and *Echinochloa crus-galli*

Petri dishes were prepared as described for Experiments 1 and 2. The filter paper used for this experiment was 73 g/m^2^. The number of seeds placed in each Petri dish and the number of replications depended on the weed tested, because of their differences in seed size and requirements for an optimal germination (verified in previous assays). For *A. retroflexus* and *P. oleracea* 20 seeds were placed on each Petri dish, and 5 replications were performed. For *E. crus-galli* and *A. fatua* 10 and 5 seeds were used respectively in each Petri dish, and with both weeds, 10 replications were performed.

Petri dishes were incubated in an EGCHS series plant growth chamber (Equitec, Madrid, Spain). According to previous assays, the germination conditions were the same for the summer weeds *P. oleracea*, *A. retroflexus* and *E. crus-galli*: 30 ± 0.1 °C, 16 h in light and 20 ± 0.1 °C, 8 h in dark, while for *A. fatua*, which is a winter–spring weed, the germination conditions were set at 23.0 ± 0.1 °C, 8 h in light and 18.0 ± 0.1 °C, 16 h in dark. TC4 was tested at the same concentrations used in Experiments 1 and 2 in all weeds (0.125, 0.25, 0.5 and 1 µL/mL), and also at 2 µL/mL on *P. oleracea* and *E. crus-galli*, as in previous assays had exhibited more resistance to EOs than other tested weeds. To evaluate the phytotoxic activity of TC4 EO on weed germination and seedling length, data were recorded after 3, 5, 7, 10 and 14 days, by registering digital images of all Petri dishes that were later processed using Digimizer v.4.6.1 (MedCalc Software, Ostend, Belgium, 2005–2016) software.

### 3.3. In Vivo Herbicidal Activity Assays

#### 3.3.1. Greenhouse Conditions during the Experiments 

**Figure 12:**
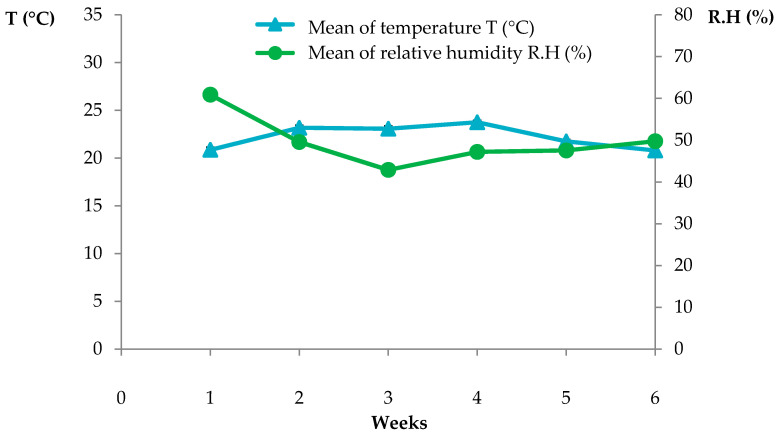
Greenhouse conditions during the experiment testing TC3 EO application directly injected into the soil or supplied with water, emulsified with Tween 20 or Fitoil.

The greenhouse used in all experiments was number 8 located in Universitat Politècnica de València (UPV) (Valencia, Spain). Table 13 and Figure 12 report the temperature and relative humidity measured in the greenhouse during the experiments. These data were acquired by a HOBO U23 Pro v2 External Temperature Data Logger (Onset Computer Corporation, Bourne, MA, USA). 

#### 3.3.2. Experiment 1—Pre-emergence Assays with TC3 against Sown Target Seeds and Weeds Contained in the Soil Seedbank in Pots under Greenhouse Conditions (2013)

1. With the objective to find the best way to apply *T. capitata* EO to the soil to control weeds in pre-emergence, in this trial, three different application methods of TC3 EO to the soil were tested, and three concentrations were used (1, 2 and 4 µL/mL) with each method:TC3 application directly injected into the soil (I1, I2 and I4)
2. Application of TC3 by irrigation, supplied with water, using Tween 20 (Sigma-Aldrich, Darmstadt, Germany) as emulsifier (T1, T2 and T4).
3. Application of TC3 by irrigation, supplied with water, using Fitoil (Xeda Italia s.r.l., Forlí, Italy) as emulsifier.

For each treatment, pots of 2 L capacity were used, filled with 3 cm of perlite at the bottom, and 6 cm of soil added on top. The soil was collected from a citrus orchard non treated with herbicides located in Puzol (Valencia province, Spain; 39°37′24.8″ N, 0°17′25.6″ W). Five repetitions were prepared for each treatment and 5 for the controls (one control for each mode of application). Ten seeds of *P. oleracea*, *A. hybridus, E. canadensis*, *S. verticillata*, *S. nigrum*, *S. oleraceus* and *C. album* were sown in each pot, covered with a small layer of soil, and then irrigated with 200 mL of water. The three concentrations of TC3 were applied the following day, using the three methods described as follows:

1. Injected directly into the soil, in the centre of the pot, using a pipette.
2. Watering each pot with the corresponding dose of TC3 for each concentration emulsified with 100 µL of Fitoil (following the recommendations of the manufacturer: 100 mL/hL) in 100 mL of water.
3. Watering each pot with the corresponding dose of TC3 for each concentration emulsified with 9.1 µL of Tween 20 (to obtain a concentration of 100 mg/L) [85] in 100 mL of water.

Once a week, the pots were irrigated to maintain the soil moisture level at the field capacity, and the weeds grown in each pot were counted and identified. When weeds were at the blooming stage they were extracted, and their fresh and dry weight were registered.

#### 3.3.3. Experiments 2 and 3—Pre- and Post-Emergence Assays with TC4 against the Spontaneous Weeds Contained in the Soil Seedbank (2015)

##### Pre-Emergence Trial

For each treatment, 3 trays of 34 × 23 × 7 cm were prepared, filling them with 1 cm of perlite at the bottom and adding a 5 cm layer of soil at the top. The soil was collected from an experimental loquat plot owned by the Cooperative of Callosa d’ En Sarrià (Alicante province, Spain), which was not treated with any herbicide during the campaign, but had been previously treated with glyphosate.

Before soil extraction, an inspection of the experimental plot was carried out, to inventory the main present weed species, which were *Amaranthus blitoides* S. Watson (the most abundant, with 40–45% coverage), *Amaranthus albus* L. (30% coverage), *Erigeron bonariensis* L. and *Euphorbia prostrata* Aiton (10–15% coverage each). Other detected species, but only as isolated individuals, were *Chenopodium album* L., *Parietaria judaica* L., *Solanum nigrum* L., *Convolvulus althaeoides* L., *Convolvulus arvensis* L., *Echinochloa colona* (L.) Link, *Sonchus oleraceus* (L.) L. and *Malva neglecta* Wallr.

Once the trays were prepared, they were irrigated with water up to 2/3 of the soil field capacity (previously calculated) and then the treatments were applied, to complete the remaining 1/3 of the soil field capacity, by irrigation, supplied with water, using Fitoil as emulsifier, at the dose recommended by the manufacturer (100 mL/hL). TC4 was tested at 1, 2 and 4 μL/mL, applied at different volumes (5.5, 2.775 and 1.83 L/m^2^), in order to find the best combination for achieving the maximum EO herbicidal effect. The applied treatments were: T1: control (irrigated with water), T2: 1 µL/mL at 5.5 L/m^2^, T3: 2 µL/mL at 5.5 L/m^2^, T4: 2 µL/mL at 2.775 L/m^2^, T5: 4 µL/mL at 2.775 L/m^2^ and T6: 4 µL/mL at 1.83 L/m^2^. This combination of treatments was focused to find out the most effective solution between the application of a higher volume of the EO solution—capable to better moisten the soil and reach more weed seeds—and the application of a lower volume of EO solution with a higher concentration—the EO being more effective against the weed seeds. 

The trial was evaluated twice a week (every 3 and 4 days) by recording images of the trays, in which the emerged plants were counted and the average height of the seedlings was measured. The images were then processed using UTHSCSA Image Tool 3.0. software. 

##### Post Emergence Trial

The pre-emergence test trays were used to perform this experiment, 24 days after the first treatment. Before the realization of the trial, the number of existing plants, their coverage area and height average were measured in each tray. The same treatments as described above were applied by spraying with a manual 1.5 L Hozelock brand sprayer. Previously, the trays had been irrigated at 2/3 of their field capacity.

The trial was evaluated by direct observation of the EO phytotoxic effects on the treated seedlings and recording images of the trays, 3 and 7 days after treatments application throughout the test, living plants were counted and their coverage area was measured. Recorded images were processed by UTHSCSA Image Tool 3.0 software. 

#### 3.3.4. Experiment 4—Post-Emergence Assays with TC4 and Carvacrol against *Avena fatua*, Applied by Irrigation and Spraying (2017)

*A. fatua* seeds described for in vitro experiment 3 were used for this trial. Their germination capability was previously verified in a germination-growth chamber from Equitec (Madrid, Spain) set at the conditions described for the in vitro bioassay. After one week, the emerged seedlings were individually transplanted in polypropylene square pots (8 × 8 × 7 cm) previously filled with a 2 cm drainage layer of perlite in the bottom (7 g) and 5 cm of soil (220 g) collected from the same citrus orchard described for experiment 1. Transplanted seedlings were transferred to the greenhouse. When the plants reached the phenological stage of 2–3 true leaves, corresponding to stage 12–13 of BBCH (Biologische Bundesanstalt, Bundessortenamt und Chemische Industrie) scale, treatments were applied: 80 mL of water were added to each pot, to bring the soil to 4/5 of its water holding capacity (WHC), and left overnight. The day after, 100% of soil WHC was reached by adding 20 mL of the corresponding treatment. TC4 and carvacrol were supplied with water, emulsified with Fitoil (as described for previous experiments) at 4 and 8 µL/mL by spraying and watering. For spraying, the same sprayer described in experiment 3 was used. Ten plants were used for each treatment; each plant was treated with 20 mL of the corresponding solution. 

**Figure 13:**
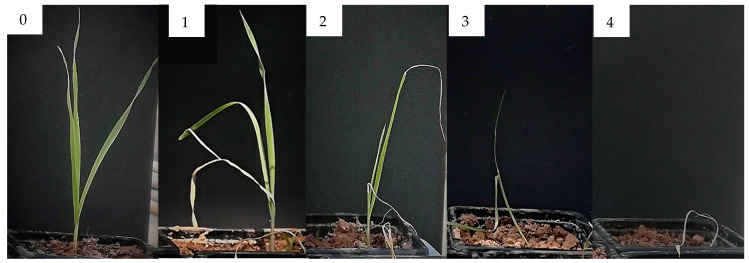
Damage level scale of *Avena fatua*. **0** Undamaged plant, **1** Plant with slight damage, **2** Plant with severe damage, **3** Critically damaged plant, **4** Dead plant.

The first evaluation was carried out 24 h after treatments application, then evaluations were done periodically (each 3 days), by registering images of the pots. At the end of the experiment, the entire plant from each pot was reclaimed by dipping in water the root apparatus to remove any soil residues and images of all plants were registered. Later, all registered images were processed using the software Digimizer v.4.6.1 (MedCalc Software, Ostend, Belgium, 2005–2016), to determine efficacy, damage level and plants length (aerial parts and roots). Efficacy, defined as the capacity to kill the plants, was rated for each plant by attributing the value 0 if the plant was alive and the value 100 if the plant was dead. Damage level ranged from 0 (no damage) to 4 (death of the plant) (Figure 13). Total length of the whole plants, and of aerial parts and roots, as well as fresh and dry weight, were also recorded.

#### 3.3.5. Experiment 5—Post-Emergence Assays with TC4 against Target Weeds *Portulaca oleracea*, *Avena fatua* and *Echinochloa crus-galli* Applied by Spraying (2018)

The same seeds described for the in vitro bioassays (experiment 3) were used in this trial. Their germination capability was verified in a germination-growth chamber from Equitec (Madrid, Spain) set in the same conditions described for the in vitro tests. After one week, the emerged seedlings were individually transplanted in polypropylene square pots, prepared as described in experiment 4. When the plants reached the phenological stage of 2–3 true leaves (stage 12-13 BBCH scale) for the monocotyledons *A. fatua* and *E. crus-galli*, and 3-4 true leaves (stage 13-14 BBCH scale) for the dicotyledon *P. oleracea*, treatments were applied by spraying as described in experiment 4. Three doses of TC4 EO were tested: 4 µL/mL, 8 µL/mL and 12 µL/mL. In addition, two controls were prepared: the first one sprayed with water (Cw) and the second sprayed with water + Fitoil at 0.5 µL/mL concentration (Cf). Ten repetition per each treatment were performed. In this experiment, the dose of Fitoil used was lower than in previous ones because a slightly stimulatory effect of Fitoil was detected in some weed species when applied by spraying (data not shown).

**Figure 14:**
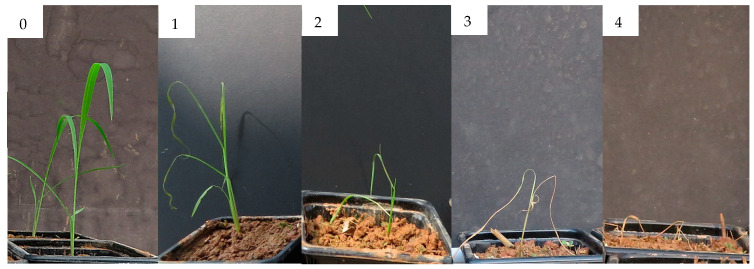
Damage level scale of *Echinochloa crus-galli*. **0** Undamaged plant, **1** Plant with slight damage, **2** Plant with severe damage, **3** Critically damaged plant, **4** Dead plant.

**Figure 15:**
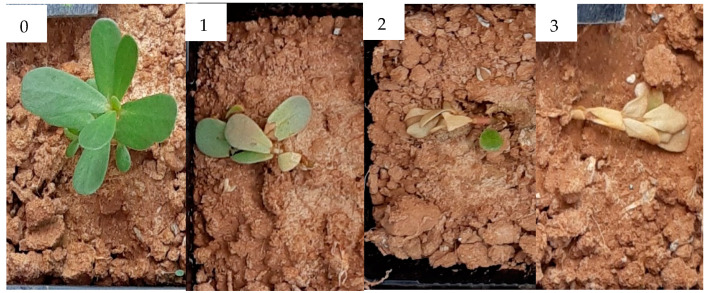
Damage level scale of *Portulaca oleracea*. **0** Undamaged plant, **1** Plant with slight damage, **2** Plant with severe damage, **3** Dead plant.

The evaluation of this trials was carried out as described for experiment 3. Figure 14 and Figure 15 report the damage level scale for *E. crus-galli* and *P. oleracea*, respectively. For *P. oleracea*, the damage level scale ranged from 0 (no damage) to 3 (dead plant). For *A. fatua* the same damage level scale used for experiment 3 was adopted.

### 3.4. Dose–Response Carvacrol Curve in Arabidopsis thaliana (L.)

Seeds of *Arabidopsis thaliana* (L.) Heynh Columbia ecotype (Col-0) were sterilized with ethanol (50%) and NaOCl (0.5%) for 3 min each, then washed in sterilized distilled water and stored in agar (0.1%) at 4 °C for 72 h. After this time, seeds were sown in square Petri dishes (100 × 15 mm) with agar medium (0.8% *w*/*v*) enriched with micro and macronutrients (0.44% Murashige-Skoog, Sigma-Aldrich, MO, USA) and supplemented with 1% sucrose [86]. Twenty-four seeds were sown in each plate under sterile conditions, and plates were kept in vertical in growth chambers at 22 ± 2 °C, with a photoperiod of 8 h light/16 h darkness, and a relative humidity of 55% for 14 days. In order to determine the phytotoxic range of carvacrol, a dose–response curve was performed for the germination and growth of *A. thaliana*. Agar solutions were prepared with a wide range of concentrations (0, 50, 100, 200, 400, 800 and 1200 µM) of carvacrol (Sigma-Aldrich, St Louis, MO, USA) using 0.1% EtOH (v:v) as solvent. Five replicates per treatment and concentration were prepared and placed in a growth chamber under controlled conditions. The germinated seeds were counted and their root length was measured 14 days after sowing (DAS).

Moreover, the morphology of carvacrol-treated radicles of *A. thaliana* was studied at 7 and 14 DAS by scanning and magnifier (Nikon SMZ 1500). Different parameters were analyzed, as the structure and root thickness, the growth direction and the morphology and abundance of root hairs, in addition to other alterations that could be related to the mode of action of carvacrol, at the cellular level. 

### 3.5. Statistical Analyses

Data were submitted to analysis of variance (ANOVA) using Statgraphics^®^ Centurion XVII (StatPoint Technologies Inc., Warrenton, VA, USA) software. Percentage values were arcsin transformed. The means were compared using Fisher’s least significant difference (LSD) test (*p* < 0.05).

A multifactorial analysis of variance (ANOVA) including species and treatments as effects was performed on efficacy data (in vivo experiment 5), followed by Fisher’s multiple comparison test (LSD intervals, Least Significant Difference, at *p* < 0.05) for the separation of the means.

## 4. Conclusions

*Thymbra capitata* EO exhibited strong herbicidal effectiveness in all described assays, showing a wide spectrum of activity: besides controlling the germination and growth of several important Mediterranean weeds, it also proved effective in preventing seed germination when applied to the soil in pre-emergence, demonstrating to be a suitable tool for sustainable weed management. Some aspects were revealed, as its activity depended on the species against it was applied, the doses, the formulation, the way of application, and the phenological stage of the treated plants. All these factors must be taken into account when planning to use this EO inside an IWM strategy. It could be used to control weeds in fruit crops as a broad spectrum herbicide, and, more selectively, to control weeds in mono or dicotyledonous crops, by managing the correct stage of application for weeds and crops and the proper mode of application, as it was observed that it was more effective when applied by irrigation in monocotyledonous species and by spraying in dicotyledonous.

## Figures and Tables

**Table 1:** Chemical composition of *Thymbra capitata* EOs tested. **TC1**, *T. capitata* from Enna (Enna province, Sicily) collected at flowering stage, **TC2**, *T. capitata* from Riesi (Caltanissetta province, Sicily) collected at vegetative stage. **TC3**, *T. capitata* from Carmona (Seville province, Spain) collected at flowering stage. **TC4**, EO purchased from Bordas, S.A. (Seville province, Spain).

Component	KI	TC1	TC2	TC3	TC4
**Monoterpene Hydrocarbons**		10.42	18.35	17.33	22.54
α-Thujene	931	-	0.86	2.10	0.89
α-Pinene	939	0.48	0.65	0.71	0.74
Camphene	954	0.19	0.33	0.09	-
β-Pinene	979	0.08	0.10	0.06	0.29
Myrcene	991	1.06	1.39	2.03	1.95
α-Phellandrene	1006	0.05	0.01	0.27	0.16
δ-3-Carene	1010	t	t	0.08	-
α-Terpinene	1017	0.64	0.55	1.37	1.61
*p*-Cymene	1026	**6.78**	**12.07**	**4.57**	**8.93**
Limonene	1030	0.17	0.38	0.23	0.20
β-Phellandrene	1036	-	-	0.24	-
*trans*-β-Ocimene	1052	-	-	0.05	-
γ-Terpinene	1060	0.97	2.01	**5.45**	**7.77**
Terpinolene	1090	t	t	0.08	
**Oxygenated Monoterpenes**		78.94	68.79	79.07	73.98
1,8-Cineole	1033	t	-	-	0.11
*cis*-Sabinene hydrate	1079	0.38	0.33	0.19	-
*trans-*Sabinene hydrate	1095	t	t	-	-
Linalool	1100	0.40	1.26	0.93	0.77
Borneol	1179	t	t	0.06	0.16
Terpinen-4-ol	1188	0.82	1.11	0.54	0.37
Cryptone	1202	-	0.04	-	-
Thymol	1292	-	-	0,22	0.27
Carvacrol	1302	**77.02**	**65.55**	**77.13**	**72.30**
Carvacrol acetate	1374	0.32	0.50	-	-
**Sesquiterpene Hydrocarbons**		4.74	8.02	2.50	3.14
β-Caryophyllene	1419	**4.42**	**6.99**	2.50	3.14
Aromadendrene	1442	-	0.10	-	-
α-Humulene	1457	-	t	-	-
*allo-*Aromadendrene	1459	-	0.04	-	-
Bicyclogermacrene	1500	-	0.21	-	-
β-Bisabolene	1507	0.24	0.68	-	-
Ƴ-Cadinene	1516	-	t	-	-
δ-Cadinene	1526	0.08	t	-	-
**Oxygenated Sesquiterpenes**		1.32	2.34		0.14
Spathulenol	1581	t	0.21	-	-
Caryophyllene oxide	1586	1.32	2.13	-	0.14
**Diterpene hydrocarbons**	-	0.11	-	-	-
Abietatriene	2072	0.11	-	-	-
**Aromatics**	-	1.77	-	-	-
Eugenol	1361	1.77	-	-	-
Chavibetol acetate	1520	t	-	-	-
**Others**		0.30	0.38	0.18	-
1-Octen-3-ol	979	0.30	0.32	0.18	-
3-Octanol	997	t	t	t	-
2-Nonanone	2100	t	0.06		
**TOTAL IDENTIFIED (%)**		**97.60**	**97.88**	**99.08**	**99.08**

**Table 2:** Phytotoxic effects of *Thymbra capitata* EOs obtained from plants at blooming (TC1) and vegetative stage (TC2) and carvacrol, on *Portulaca oleracea* and *Erigeron canadensis* seed germination.

Seed Germination (%)			
	Concentration µL/mL	*Portulaca oleracea*	*Conyza canadensis*
**TC1**	0 (control)	87.0 ± 1.2 a	94.0 ± 2.4 a
	0.125	46.0 ± 1.4 b	0.0 ± 0.0 b
	0.250	7.0 ± 3.7 c	0.0 ± 0.0 b
	0.5	0.0 ± 0.0 c	0.0 ± 0.0 b
	1	0.0 ± 0.0 c	0.0 ± 0.0 b
**TC2**	0 (control)	87.0 ± 1.2 a	94.0 ± 2.4 a
	0.125	39.0 ± 9.3 b	0.0 ± 0.0 b
	0.250	0.0 ± 0.0 c	0.0 ± 0.0 b
	0.5	0.0 ± 0.0 c	0.0 ± 0.0 b
	1	4.0 ± 4.0 c	0.0 ± 0.0 b
**Carvacrol**	0 (control)	88.8 ± 5.5 a	95.0 ± 2.2 a
	0.125	0.0 ± 0.0 b	0.0 ± 0.0 b
	0.250	0.0 ± 0.0 b	0.0 ± 0.0 b
	0.5	0.0 ± 0.0 b	0.0 ± 0.0 b
	1	0.0 ± 0.0 b	0.0 ± 0.0 b

Values are means ± standard error of 5 replicates, 20 seeds each, after 14 days of incubation. Within each species, for each treatment (TC1, TC2 or carvacrol), different letters in the same column indicate significant differences among concentrations (*p* < 0.05) using Fisher’s least significant difference (LSD) test.

**Table 3:** Phytotoxic effects of *Thymbra capitata* EO (TC3) on *Solanum nigrum*, *Chenopodium album*, *Sonchus oleraceus* and *Setaria verticillata* seed germination.

Seed Germination (%)				
Concentration µL/mL	*Solanum nigrum*	*Chenopodium album*	*Sonchus oleraceus*	*Setaria verticillata*
0 (control)	99.00 ± 1.00 a	98.00 ± 2.00 a	83.00 ± 4.4 a	95.00 ± 1.60 a
0.125	26.00 ± 11.1 b	0.00 ± 0.00 b	0.00 ± 0.00 b	19.00 ± 7.50 b
0.250	24.00 ± 19.4 bc	0.00 ± 0.00 b	0.00 ± 0.00 b	13.00 ± 6.00 b
0.5	0.00 ± 0.00 c	0.00 ± 0.00 b	0.00 ± 0.00 b	0.00 ± 0.00 c
1	0.00 ± 0.00 c	0.00 ± 0.00 b	0.00 ± 0.00 b	0.00 ± 0.00 c

Values are means ± standard error of 5 replicates, 20 seeds each, after 14 days of incubation. Within each species, different letters in the same column indicate significant differences among concentrations (*p* < 0.05) using Fisher’s least significant difference (LSD) test.

**Table 4:** Phytotoxic effects of *Thymbra capitata* EO (TC3) on *Solanum nigrum*, *Chenopodium album*, *Sonchus oleraceus* and *Setaria verticillata* seedling length.

Seedling Length (mm)				
Concentration µL/mL	*Solanum nigrum*	*Chenopodium album*	*Sonchus oleraceus*	*Setaria verticillata*
0 (control)	47.07 ± 1.58 a	24.71 ± 1.19 a	17.23 ± 1.48 a	65.00 ± 2.22 a
0.125	12.40 ± 4.16 b	-	-	19.94 ± 6.15 b
0.250	2.46 ± 2.46 bc	-	-	16.23 ± 4.97 b
0.5	-	-	-	-
1	-	-	-	-

Values are means ± standard error of 5 replicates of 20 seeds each after 14 d of incubation. Within each species, different letters in the same column indicate that means among concentrations are different (*p* < 0.05) using Fisher’s least significant difference (LSD) test.

**Table 5:** Phytotoxic effects of *Thymbra capitata* EO (TC4) on *Amaranthus retroflexus*, *Portulaca oleracea*, *Avena fatua* and *Echinochloa crus-galli* seed germination.

Seed Germination (%)				
Concentration µL/mL	*Amaranthus retroflexus*	*Portulaca oleracea*	*Avena fatua*	*Echinochloa crus-galli*
0 (control)	87.0 ± 2.0 a	76.0 ± 5.8 a	64.0 ± 5.8 a	89.0 ± 3.5 a
0.125	62.0 ± 6.8 b	26.0 ± 4.8 b	56.0 ± 9.3 a	-
0.250	18.0 ± 1.2 c	17.0 ± 4.1 bc	14.0 ± 7.9 b	50.0 ± 5.6 b
0.5	6.0 ± 2.9 d	9.0 ± 2.9 c	0.0 ± 0.0 c	31.0 ± 6.7 c
1	0.0 ± 0.0 e	2.0 ± 1.2 d	0.0 ± 0.0 c	13.0 ± 4.0 d
2	-	0.0 ± 0.0 d	-	0.0 ± 0.0 e

Values are means ± standard error of 5 replicates with 20 seeds for dicotyledons and 10 replicates for monocotyledons, with 10 seeds for *E. crus-galli* and 5 seeds for *A. fatua* after 14 d of incubation. Within each species, different letters in the same column indicate that means are different among concentrations (*p* < 0.05) using Fisher’s least significant difference (LSD) test.

**Table 6:** Phytotoxic effects of *T. capitata* EO (TC4) on *Amaranthus retroflexus*, *Portulaca oleracea*, *Avena fatua* and *Echinochloa crus-galli* coleoptile and radical length.

Coleoptile and Radicle Length (mm)				
Concentration µL/mL	*A. fatua* Coleoptile Length	*A. fatua* Radicle Length	*E. crus-galli* Coleoptile Length	*E. crus-galli* Radicle Length
0 (control)	22.52 ± 0.78 a	21.12 ± 1.23 a	51.28 ± 1.88 a	38.81 ± 1.12 a
0.125	6.84 ± 1.43 b	1.55 ± 0.37 b	-	-
0.250	1.11 ± 0.48 c	0.39 ± 0.11 b	26.03 ± 1.59 b	18.00 ± 2.28 b
0.5	-	-	22.56 ± 1.84 b	14.60 ± 1.66 b
1	-	-	15.50 ± 0.65 c	12.70 ± 0.60 b
2	-	-	-	-

Values are means ± standard error of ten replicates, with 10 seeds for *E. crus-galli* and 5 seeds for *A. fatua* after 14 days of incubation. Within each species different letters in the same column indicate that means are different among concentrations (*p* < 0.05) using Fisher’s least significant difference (LSD) test.

**Table 7:** TC4 efficacy per species and per treatment. T4, T8, T12: 4, 8, 12 µL/mL EO; Cw: water control; Cf: Fitoil control.

**Species**	**Efficacy**
*Portulaca oleracea*	60.00 ± 4.06 a
*Avena fatua*	32.00 ± 4.06 b
*Echinochloa crus-galli*	00.00 ± 4.06 c
**Treatments**	**Efficacy**
Cw	0.00 ± 5.24 c
Cf	0.00 ± 5.24 c
T4	33.33 ± 5.24 b
T8	53.33 ± 5.24 a
T12	66.66 ± 5.24 a

Mean values of ten replicates ± standard error. Different letters indicate statistical differences among species or treatments (*p* < 0.05) using Fisher’s least significant difference (LSD) test.

**Table 8:** Efficacy, effects on several plant traits (aerial part, root and total length, fresh and dry weight), and damage level of TC4 EO at different application doses on *P. oleracea*. T4, T8, T12: 4, 8, 12 µL/mL EO; Cw: water control; Cf: Fitoil control.

Treatments	Efficacy	Aerial Part Length (cm)	Root Length (cm)	Total Length (cm)	Fresh Weight (g)	Dry Weight (g)	Damage Level
Cw	0.00 b	6.55 b	13.09 a	19.64 b	1.23 a	0.15 a	0.00 b
Cf	0.00 b	7.39 a	13.87 a	21.26 a	1.30 a	0.17 a	0.00 b
T4	100.00 a	0.00 c	0.00 b	0.00 c	0.00 b	0.00 b	3.00 a
T8	100.00 a	0.00 c	0.00 b	0.00 c	0.00 b	0.00 b	3.00 a
T12	100.00 a	0.00 c	0.00 b	0.00 c	0.00 b	0.00 b	3.00 a

Mean values of ten replicates. Different letters in the same column indicate statistical differences (*p* < 0.05) using Fisher’s least significant difference (LSD) test.

**Table 9:** Efficacy, effects on several plant traits (aerial part, root and total length, fresh and dry weight), and damage level of TC4 EO at different application doses on *A. fatua*. T4, T8, T12: 4, 8, 12 µL/mL EO; Cw: water control; Cf: Fitoil control.

Treatments	Efficacy	Aerial Part Length (cm)	Root Length (cm)	Total Length (cm)	Fresh Weight (g)	Dry Weight (g)	Damage Level
Cw	0.00 c	20.16 a	14.57 ab	34.74 a	0.58 a	0.07 a	0.20 c
Cf	0.00 c	21.74 a	16.02 a	37.76 a	0.76 a	0.07 a	0.30 c
T4	0.00 c	14.77 b	11.16 b	25.93 b	0.44 ab	0.05 a	1.80 b
T8	60.00 b	3.53 c	3.99 c	7.35 c	0.09 bc	0.02 b	3.50 a
T12	100.00 a	0.00 c	0.00 d	0.00 d	0.00 c	0.00 b	4.00 a

Mean values of ten replicates. Different letters in the same column indicate statistical differences (*p* < 0.05) using Fisher’s least significant difference (LSD) test.

**Table 10:** Efficacy, effects on several plant traits (aerial part, root and total length, fresh and dry weight), and damage level of TC4 EO at different application doses on *E. crus-galli*. T4, T8, T12: 4, 8, 12 µL/mL EO; Cw: water control; Cf: Fitoil control.

Treatments	Efficacy	Aerial Part Length (cm)	Root Length (cm)	Total Length (cm)	Fresh Weight (g)	Dry Weight (g)	Damage Level
Cw	0.00	27.19 a	20.17 a	47.37 a	1.43 a	0.15 a	0.00 d
Cf	0.00	26.80 a	20.07 a	46.88 a	1.42 a	0.15 ab	0.10 d
T4	0.00	25.15 ab	19.97 a	45.12 a	1.12 a	0.12 abc	0.60 c
T8	0.00	23.46 b	18.14 a	42.60 a	1.08 a	0.12 bc	1.00 b
T12	0.00	18.39 c	14.39 b	32.78 b	0.95 b	0.10 c	1.90 a

Mean values of ten replicates. Different letters in the same column indicate statistical differences (*p* < 0.05) using Fisher’s least significant difference (LSD) test.

**Table 11:** Efficacy of TC4 on target weed species *A. fatua*, *E. crus-galli*, *E. bonariensis* and *P. oleracea* applied by irrigation or spraying. (CW—water control, CF—Fitoil control, TC4—TC4 at 4 µL/mL, TC8—TC4 at 8 µL/mL and TC12—TC4 at 12 µL/mL).

***Avena fatua***		
**Treatment**	**Irrigation**	**Spraying**
CW	0.00 ± 0.00 c	0.00 ± 0.00 c
CF	0.00 ± 0.00 c	0.00 ± 0.00 c
TC4	80.00 ± 13.33 b	0.00 ± 0.00 c
TC8	90.00 ± 9.99 ab	60.00 ± 7.83 b
TC12	100.00 ± 0.00 a	100.00 ± 0.00 a
***Echinochloa crus-galli***		
**Treatment**	**Irrigation**	**Spraying**
CW	0.00 ± 0.00 c	0.00 ± 0.00
CF	0.00 ± 0.00 c	0.00 ± 0.00
TC4	10.00 ± 9.99 c	0.00 ± 0.00
TC8	50.00 ± 16.66 b	0.00 ± 0.00
TC12	100.00 ± 0.00 a	0.00 ± 0.00
***Portulaca oleracea***		
**Treatment**	**Irrigation**	**Spraying**
CW	0.00 ± 0.00 c	0.00 ± 0.00 b
CF	0.00 ± 0.00 c	0.00 ± 0.00 b
TC4	0.00 ± 0.00 c	100.00 ± 0.00 a
TC8	40.00 ± 16.32 b	100.00 ± 0.00 a
TC12	90.00 ± 9.99 a	100.00 ± 0.00 a
***Erigeron bonariensis***		
**Treatment**	**Irrigation**	**Spraying**
CW	0.00 ± 0.00 b	0.00 ± 0.00 c
CF	0.00 ± 0.00 b	0.00 ± 0.00 c
TC2	10.00 ± 10.00 b	100.00 ± 0.00 a
TC4	70.00 ± 15.30 a	80.00 ± 13.30 b
TC8	90.00 ± 10.00 a	100.00 ± 0.00 a

Mean values ± standard errors of ten replicates. For each species, different letters in the same column indicate statistically significant differences between treatments (*p* < 0.05) using Fisher’s least significant difference (LSD) test.

**Table 12:** Efficacy, effects on several plant traits (aerial part, root and total length, fresh and dry weight), and damage level of TC4 EO (TC) and carvacrol (CV), applied at 4 and 8 µL/mL, by irrigation (CVR4, CVR8; TCR4, TCR8) and spraying (CVP4, CVP8; TCP4, TCP8) on *Avena fatua* (WCR: irrigated water control; WCP: sprayed water control).

**Treatments Applied by Irrigation**						
**Treatment**	**Efficacy**	**Aerial Part Length (cm)**	**Root Length (cm)**	**Fresh Weight (g)**	**Dry Weight (g)**	**Damage Level**
WCR	0.00 c	16.42 a	16.10 a	0.60 a	0.15 a	1.00 c
CVR4	80.00 a	1.94 c	0.89 c	0.00 bc	0.00 bc	2.40 ab
CVR8	90.00 a	0.99 c	1.13 c	0.02 bc	0.00 c	2.70 ab
TCR4	30.00 b	7.63 b	4.87 b	0.09 b	0.03 b	2.00 b
TCR8	100.00 a	0.00 c	0.00 c	0.00 c	0.00 c	3.00 a
**Treatments Applied by Spraying**						
**Treatment**	**Efficacy**	**Aerial Part Length (cm)**	**Root Length (cm)**	**Fresh Weight (g)**	**Dry Weight (g)**	**Damage Level**
WCP	0.00 b	17.56 a	16.07 a	0.21 a	0.10 ab	1.00 bc
CVP4	10.00 b	14.76 ab	8.08 b	0.17 a	0.13 a	0.30 c
CVP8	20.00 ab	12.20 ab	8.50 b	0.20 a	0.10 ab	1.40 b
TCP4	30.00 ab	9.45 bc	6.27 b	0.18 a	0.05 ab	1.50 b
TCP8	50.00 a	5.03 c	4.98 b	0.04 b	0.02 b	2.60 a

Mean values of ten replicates. For each mode of application, different letters in the same column indicate statistically significant differences (*p* < 0.05) using Fisher’s least significant difference (LSD) test.

**Table 13:** Temperature and relative humidity conditions (Mean, Maximum (Max.) and Minimum (Min.)) in the greenhouse during the experiments.

Trial	Starting-End Date	Temperature (°C)			Relative Humidity (%)		
		Mean	Max.	Min.	Mean	Max.	Min.
TC3 injected into the soil/TC3+Tween20/TC3+ Fitoil	27/02/2013–10/04/2013	22.3	34.9	15.8	49.4	92.8	14.4
TC4 in pre- and post-emergence applied to weeds from soil seedbank	27/7/2015–27/8/2015	29.1	33.9	18.7	-	-	-
TC4 and carvacrol (4, 8 μL/mL) irrigation, spray on *A. fatua*	01/05/2017–24/05/2017	26.1	35.2	20.1	66.9	85.6	45.8
TC4 (4,8,12 μL/mL) spray on *P. oleracea*	10/08/2018–30/08/2018	25.4	36.3	18.6	59.3	81.1	29.8
TC4 (4,8,12 μL/mL) spray on *A. fatua*	02/05/2018–31/05/2018	24.8	35.4	19.1	51.7	82.1	21.6
TC4 (4,8,12 μL/mL) spray on *E. crus galli*	26/09/2018–05/10/2018	24.1	36.3	18.8	56.0	88.5	25.5

Relative humidity data of experiment 2 are not reported because the data logger did not register them.

## Supplementary Materials

The following are available online: Tables S8–S12.
