# Supplementary material for: Herbicidal Activity of Thymbra capitata (L.) Cav. Essential Oil
Source: Molecules. 2020 Jun 19;25(12):2832. doi: 10.3390/molecules25122832 (PMC7357079; doi:10.3390/molecules25122832)
Supplement: Supplementary file 1 [file molecules-25-02832-s001.pdf]

Table S8. Effects of TC4 EO (T4, T8 and T12 are 4, 8 and 12 μL/mL dose of application, Cw is control water and Cf control Fitoil) on the efficacy, plant biometric variables (aerial part, root and total length, fresh and dry weight) and damage level of *P. oleracea*.

Treatment	Efficacy	Aerial part lenght (cm)	Root lenght (cm)	Total lenght (cm)	Fresh weight (g)	Dry weight (g)	Damage level
**Cw**	0.00 ± 0.00 b	6.55 ± 0.38 b	13.09 ± 1.02 a	19.64 ± 1.31 a	1.23 ± 0.12 a	0.15 ± 0.01 a	0.00 ± 0.00 b
**Cf**	0.00 ± 0.00 b	7.39 ± 0.33 a	13.87 ± 0.66 a	21.26 ± 0.72 a	1.30 ± 0.09 a	0.17 ± 0.02 a	0.00 ± 0.00 b
**T4**	100.00 ± 0.00 a	0.00 ± 0.00 c	0.00 ± 0.00 b	0.00 ± 0.00 b	0.00 ± 0.00 b	0.00 ± 0.00 b	3.00 ± 0.00 a
**T8**	100.00 ± 0.00 a	0.00 ± 0.00 c	0.00 ± 0.00 b	0.00 ± 0.00 b	0.00 ± 0.00 b	0.00 ± 0.00 b	3.00 ± 0.00 a
**T12**	100.00 ± 0.00 a	0.00 ± 0.00 c	0.00 ± 0.00 b	0.00 ± 0.00 b	0.00 ± 0.00 b	0.00 ± 0.00 b	3.00 ± 0.00 a

Reported results are means ± standard error of ten replicates. Different letters in the same column indicate statistical differences at $p < 0.05$.

Table S9. Effects of TC4 EO (T4, T8 and T12 are 4, 8 and 12 μL/mL dose of application, Cw is control water and Cf control Fitoil) on the efficacy, plant biometric variables (aerial part, root and total length, fresh and dry weight) and damage level of A. *fatua*

Treatment	Efficacy	Aerial part lenght (cm)	Root lenght (cm)	Total lenght (cm)	Fresh weight (g)	Dry weight (g)	Damage level
**Cw**	0.00 ± 0.00c	20.16 ± 1.11 a	14.57 ± 0.78 ab	34.74 ± 1.44 a	0.58 ± 0.23 a	0.07 ± 0.01 a	0.20 ± 0.13 c
**Cf**	0.00 ± 0.00 c	21.74 ± 1.55 a	16.02 ± 0.93 a	37.76 ± 2.11 a	0.76 ± 0.14 a	0.07 ± 0.01 a	0.30 ± 0.15 c
**T4**	0.00 ± 0.00 c	14.77 ± 1.79 b	11.16 ± 1.16 b	25.93 ± 2.60 b	0.44 ± 0.13 ab	0.05 ± 0.01 a	1.80 ± 0.29 b
**T8**	60.00 ± 16.33 b	3.53 ± 1.62 c	3.99 ± 1.77 c	7.53 ± 3.23 c	0.09 ± 0.04 bc	0.02 ± 0.01 b	3.50 ± 0.22 a
**T12**	100.00 ± 0.00 a	0.00 ± 0.00 c	0.00 ± 0.00 d	0.00 ± 0.00 d	0.00 ± 0.00 c	0.00 ± 0.00 b	4.00 ± 0.00 a

Reported results are means ± standard error of ten replicates. Different letters in the same column indicate statistical differences at $p < 0.05$.

Table S10. Effects of TC4 EO (T4, T8 and T12 are 4, 8 and 12 μL/mL dose of application, Cw is control water and Cf control Fitoil) on the efficacy, plant biometric variables (aerial part, root and total length, fresh and dry weights) and damage level of *E. crus-galli*

Treatment	Efficacy	Aerial part lenght (cm)	Root lenght (cm)	Total lenght (cm)	Fresh weight (g)	Dry weight (g)	Damage level
**Cw**	0.00 ± 0.00	27.19 ± 0.86 a	20.17 ± 1.01 a	47.37 ± 1.55 a	1.43 ± 0.10 a	0.15 ± 0.01 a	0.00 ± 0.00 d
**Cf**	0.00 ± 0.00	26.80 ± 0.56 a	20,07 ± 1.32 a	46.88 ± 1.72 a	1.42 ± 0.10 a	0.15 ± 0.02 ab	0.10 ± 0.10 d
**T4**	0.00 ± 0.00	25.15 ± 1.12 ab	19.97 ± 1.20 a	45.12 ± 1.96 a	1.12 ± 0.13 a	0.12 ± 0.01 abc	0.60 ± 0.16 c
**T8**	0.00 ± 0.00	23.46 ± 1.06 b	18.14 ± 1.29 a	42.60 ± 1.66 a	1.08 ± 0.06 a	0.12 ± 0.01 bc	1.00 ± 0.00 b

**T12**	0.00 ± 0.00	18.39 ± 0.86 c	14.39 ± 0.82 b	32.78 ± 1.61 b	0.95 ± 0.04 b	0.10 ± 0.01 c	1.90 ± 0.23 a

Reported results are means ± standard error of ten replicates. Different letters in the same column indicate statistical differences at $p < 0.05$.

Table S12. Effects of TC4 EO (TC) and carvacrol (CV) at doses 4 and 8 µL/mL applied by irrigation (CVR4, CVR8; TCR4, TCR8) and spraying (CVP4, CVP8; TCP4, TCP8) (WCR is water control irrigated and WCP water control sprayed) on the efficacy, plant biometric variables (aerial part, root and total length, fresh and dry weight) and damage level of *Avena fatua*.

**Treatments applied by irrigation**						
**Treatment**	**Efficacy**	**Aerial part length (cm)**	**Root length (cm)**	**Fresh weight (g)**	**Dry weight (g)**	**Damage level**
WCR	0.00 ± 0.00 c	16.42 ± 0.56 a	16.10 ± 1.26 a	0.60 ± 0.05 a	0.15 ± 0.01 a	1.00 ± 0.00 c
CVR4	80.00 ± 13.33 a	1.94 ± 1.31 c	0.89 ± 0.59 c	0.00 ± 0.00 bc	0.00 ± 0.00 bc	2.40 ± 0.40 ab
CVR8	90.00 ± 10.00 a	0.99 ± 0.99 c	1.13 ± 1.13 c	0.02 ± 0.02 bc	0.00 ± 0.00 c	2.7 0± 0.30 ab
TCR4	30.00 ± 15.3 b	7.63 ± 2.1 b	4.87 ± 1.5 b	0.09 ± 0.00 b	0.03 ± 0.00 b	2.00 ± 0.30 b
TCR8	100.00 ± 0.00 a	0.00 ± 0.00 c	0.00 ± 0.00 c	0.00 ± 0.00 c	0.00 ± 0.00 c	3.00 ± 0.00 a
**Treatments applied by spraying**						
**Treatment**	**Efficacy**	**Aerial part length (cm)**	**Root length (cm)**	**Fresh weight (g)**	**Dry weight (g)**	**Damage level**
WCP	0.00 ± 0.00 b	17.56 ± 0.97 a	16.07 ± 1.05 a	0.21 ± 0.02 a	0.10 ± 0.00 ab	1.00 ± 0.00 bc
CVP4	10.00 ± 10.00 b	14.76 ± 2.04 ab	8.08 ± 1.28 b	0.17 ± 0.02 a	0.13 ± 0.03 a	0.30 ± 0.30 c
CVP8	20.00 ± 13.30 ab	12.20 ± 2.30 ab	8.50 ± 1.87 b	0.20 ± 0.00 a	0.10 ± 0.00 ab	1.40 ± 0.30 b
TCP4	30.00 ± 15.30 ab	9.45 ± 2.20 bc	6.27 ± 1.70 b	0.18 ± 0.00 a	0.05 ± 0.00 ab	1.50 ± 0.04 b
TCP8	50.10 ± 16.63 a	5.03 ± 2.11 c	4.98 ± 2.15 b	0.04 ± 0.02 b	0.02 ± 0.01 b	2.60 ± 0.16 a

Reported results are means ± standard error of ten replicates. For each mode of application, different letters in the same column indicate statistical differences at $p < 0.05$.
